# Encourage Sustainability by Giving Credit for Marine Protected Areas in Seafood Certification

**DOI:** 10.1371/journal.pbio.1001730

**Published:** 2013-12-10

**Authors:** Sarah E. Lester, Christopher Costello, Andrew Rassweiler, Steven D. Gaines, Robert Deacon

**Affiliations:** 1 Marine Science Institute, University of California Santa Barbara, Santa Barbara, California, United States of America; 2 Bren School of Environmental Science and Management, University of California Santa Barbara, Santa Barbara, California, United States of America; 3 Department of Economics, University of California Santa Barbara, Santa Barbara, California, United States of America

## Abstract

Sarah Lester et al. argue that sustainable seafood certification programs are missing a critical opportunity by failing to provide credit for marine protected areas. Fixing this oversight will improve the leverage of certification programs to drive sustainable fishing, with significant benefits to fish and fisheries.

Widespread concern over global fish stocks has prompted an increase in research and initiatives aimed at rebuilding ailing fisheries and incentivizing sustainable fishing practices. This promising focus on solutions coincides with a burgeoning consumer and retailer demand for environmentally friendly products (Figure 1). Sustainability certification, labeling, and consumer guides (e.g., Marine Stewardship Council, Fair Trade, Seafood Watch, etc.) are signals that help eco-minded consumers identify products that meet their standards. Accurate signals offer an immense opportunity to incentivize sustainability, increasing demand and profits for sustainable producers. Yet, while the growing number of seafood certification programs and consumer seafood guides fuel and inform demand, the pace of change is slow. One key barrier to progress is the significant lag between the implementation of reforms and the recovery of fish stocks. Without preemptive credits within certification protocols for conservation actions that can be expected to benefit the stock over time, the incentives for reforms may be limited.

**Figure 1:**
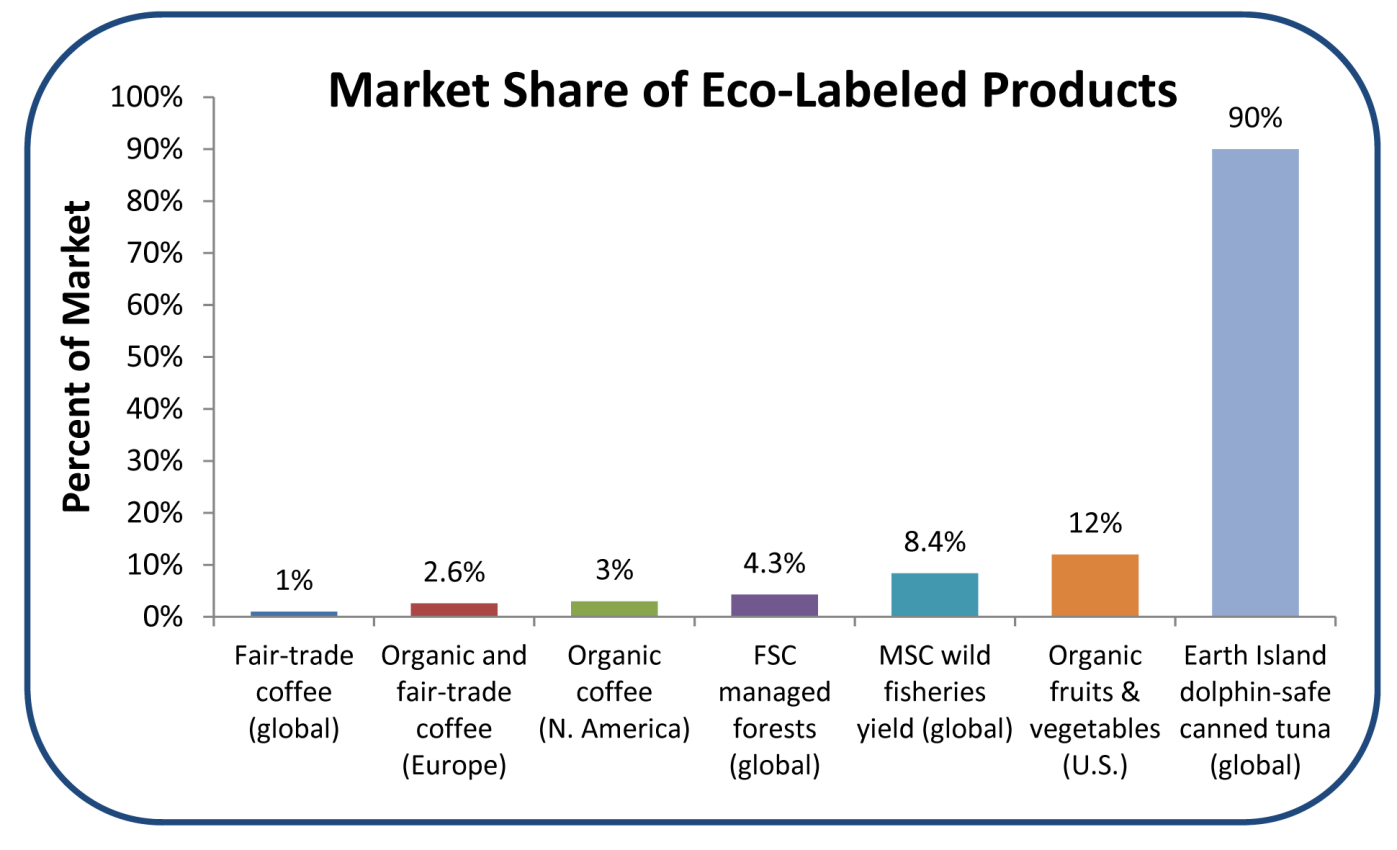
Market share of several certified products throughout various regions of the world. Included are: the percentage of global wild-capture fisheries yield that is MSC certified [14],[15], the market share of fruits and vegetables in the U.S. that are certified organic [16], the market share of coffee in Europe that is organic and fair-trade certified, the market share of coffee in North America that is certified organic, the global market share of coffee that is fair trade certified [17], the global market share of canned tuna that is Earth Island dolphin-safe certified [18], and the global percentage of forests managed according to Forest Stewardship Council standards [19],[20].

Many potential conservation reforms could qualify for preemptive credit, but the most conspicuous absence is the lack of credit for marine protected areas (MPAs). Marine protected areas are one of the most important tools for conserving the ocean's ecosystems. MPAs, similar to protected areas on land, are locations where regulations prohibit specific human activities. There has been considerable research about the effects of MPAs, particularly focused on a common class of MPAs in which all fishing is prohibited (no-take marine reserves). Studies from more than 120 no-take marine reserves around the world consistently reveal the conservation benefits of this type of protection, including higher total biomass, abundance, and average size of fish within reserves [1] (Figure 2). The data from these many existing MPAs, combined with improving spatial fisheries models, allow us to predict an MPA's future conservation benefits long before they are realized. Such empirically based model forecasts could be used to assign preemptive credit for MPAs within certification frameworks when the MPA is implemented. Doing so would reward existing MPAs for their contributions to sustainable fisheries and would provide more powerful short-term incentives to create well-designed new MPAs.

**Figure 2:**
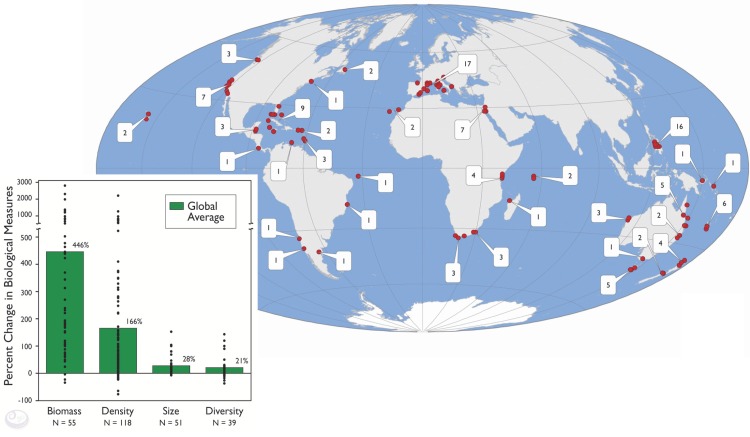
Map showing the locations of 124 marine reserves that have been studied by scientists with the results published in scientific journals. The graph shows the average changes (green bars) in fishes, invertebrates, and seaweeds within these 124 marine reserves. Although changes varied among reserves (black dots), most reserves had positive changes. Data from [1]. Graphics reproduced with permission from the Partnership for Interdisciplinary Studies of Coastal Oceans [21].

## Why We Need to Marry Seafood Certification and MPAs

Given growing green demand, the time is ripe for seafood certification to drive global fishery sustainability. Yet many existing programs are criticized for not being up to this challenge [2],[3]. Consider the example of the most prominent global fishery certifier, the Marine Stewardship Council (MSC). Although MSC has achieved significant market penetration with more than 200 certified fisheries (Figure 1), these tend to be large-scale fisheries in developed nations [4]. The intensive data requirements and high cost of assessment makes MSC certification inaccessible to a much broader range of sustainable fisheries [5].

There are two key consequences of these barriers to certification. First, many fisheries worldwide may already meet sustainability criteria but do not receive market recognition because they are simply too data-poor or cash-poor to seek MSC certification. Second, countless other fisheries might be induced to adopt sustainable practices if some of the practical hurdles to certification could be reduced without compromising sustainability standards. MSC has attempted to lower some of these hurdles recently. Yet neither the MSC, nor any other major seafood certifier, provides explicit credit for MPAs despite their growing global use.

There is strong scientific evidence that MPAs achieve conservation goals, particularly when they close areas to all fishing [1]. There is also evidence that, if designed well, MPAs may be able to increase the resilience of surrounding fisheries and enhance local catches [6],[7]. The magnitude and timing of fisheries benefits are less certain than the conservation benefits, because they depend on both the response of the fish inside the MPA and the responses of fishers outside the MPA to the new regulations. Nonetheless, despite the greater uncertainty, forecasts of fisheries benefits and costs from bioeconomic models have now been used successfully in MPA planning processes [8] and provide a framework for projecting benefits in certification systems. Furthermore, regardless of uncertainty regarding fishery impacts, the scientific evidence for the conservation and sustainability benefits of MPAs are sufficient to justify incorporating credit for MPAs into certification protocols. MPAs, when adequately sized relative to the adult movement potential of the target species, will reliably result in increased biomass inside of the protected area. This can help ensure safe levels of the stock and can mitigate ecosystem impacts of fishing by shielding areas from destructive fishing practices. Nearshore fisheries targeting species that have more limited movement (e.g., reef-associated species) are obvious candidates because these species respond positively to protection even by small MPAs, but all fisheries are potential candidates given a sufficiently sized and well-enforced protected area.

Sustainability standards, including those of MSC and the Monterey Bay Aquarium's Seafood Watch program, tend to evaluate fisheries based on three core tests: (1) Are harvesting pressure and fish stocks at safe levels? (2) Are fishing practices that have a significant negative impact on the ecosystem prohibited? and (3) Is an effective management system in place [9]? We argue that if explicitly incorporated into certification, MPAs would help fisheries pass each of these tests. First, MPAs protect some of the target stock from fishing. Second, MPAs protect habitat and non-target species from fishing impacts. Third, MPAs can reduce the complexity of required management institutions. If the MPA is well enforced and protects enough of the stock, active management outside may be less critical; effective enforcement of the MPA may be sufficient to ensure stock sustainability. MPAs act as a simple and cost-effective insurance policy against intense fishing and mismanagement in adjacent waters.

If certification schemes were to provide explicit credit for MPAs, certification would become a possibility for a wider range of small-scale, data-poor, and cash-poor fisheries. A stock's healthy status can be verified and ensured to persist without expensive stock assessments, and developing nations that have invested in fishery conservation measures can be rewarded for their efforts. MPA creation will help associated fisheries gain a competitive advantage from certification, providing further incentives for MPA adoption by regulators and fishing interests. This is critical because many fishermen, particularly in developing countries, lack incentives to support MPAs because they cannot afford to wait for the recovery of fish stocks and the accrual of the associated fisheries benefits from MPAs. Pre-emptive credit for MPAs within certification programs, on the other hand, could provide an immediate reward through higher prices and access to new markets.

## How to Provide Credit

Despite the role that MPAs play in sustainability, we are unaware of any sustainability rating that formally and quantitatively assigns credit for establishing MPAs adjacent to fisheries. We highlight three areas of analytical innovation that can improve certification efforts now and allow them to incorporate the benefits of MPAs.

First, the contribution of MPAs to fishery sustainability can be evaluated even in the absence of sufficient information to conduct a stock assessment. Such stock assessments are quantitative analyses of a fished population; they form the backbone of most seafood certifications, but require considerable data and time to conduct. The vast majority of the world's fisheries are too small and/or impoverished to finance such assessments. For these stocks, certification currently cannot provide an incentive for sustainable harvest levels or conservation actions. Of course, MPAs do conserve fish populations in these data-poor situations, provided there is adequate enforcement. In fact, for all stocks, there is an MPA network large enough to guarantee sustainability even in the absence of any other management or detailed information on stock status. And, more commonly, a moderately sized MPA can compensate for deficiencies in management or data. Similar arguments can be made for quantifying and crediting MPAs for their protection of the broader ecosystem.

Second, formal stock assessment methods must be revised to account for fish biomass within MPAs. Traditional stock assessments typically ignore the population of fish inside of the MPAs, substantially undervaluing the role of MPAs in maintaining the fish population as a whole and undermining support for their use as a management tool. For example, consider a hypothetical case where a fishery seeking certification decides to protect 20% of the region in MPAs, prohibiting fishing in these areas. In an assessment that ignores the MPA, a smaller portion of the population is now assessed (encompassing the 80% of the region where fishing is still allowed); the assessment will focus on the fact that populations outside of the MPAs have not increased and will overlook the healthy populations within the MPAs. Fortunately, as the scale and extent of MPA protection increases in many regions of the world's oceans, this shortcoming of stock assessments has been noted [10]. State-of-the-art stock assessment science includes novel approaches to account for the biomass within MPAs [11]–[13]. In data-rich fisheries, where stock assessments are possible, application of these improved methods will inherently give appropriate sustainability credit for MPAs.

**Figure 3:**
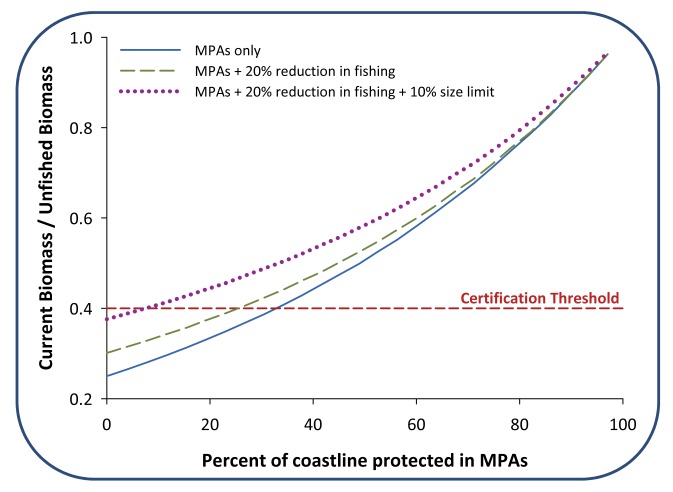
Bioeconomic models can be used to compare different strategies for achieving seafood sustainability. In this example based on the sheephead fishery in Southern California, we assume that one requirement of the certification scheme is for the biomass of the target species to be at least 40% of unfished biomass. With MPAs alone, biomass is predicted to equilibrate at certifiable levels if one third of the coastline is closed to fishing (blue solid curve). If effort is reduced by 20%, certification can be achieved by closing only one quarter of the coastline to fishing (green dashed curve). If that reduction in effort is accompanied by a 10% increase in the size limit, less than one tenth of the coastline need be closed to raise the population to 40% of its unfished biomass (purple dotted curve). Reducing fishing effort by 20% and raising the size limit is not sufficient for achieving certification without at least some MPA protection, but a mix of more conservative non-spatial regulations could achieve certification without any MPAs (not shown). See Text S1 for additional methods.

Third, we can apply predictive modeling to assess alternative routes to certification. Spatial models can be used to forecast the impacts of alternative pathways to certification and identify the most efficient combination. For example, recently developed spatial models of fish population dynamics, coupled with oceanographic models predicting movement of young fish and fleet models describing fishermen behavior, can forecast the long-term impacts on sustainable stock levels and fishery profit of different management actions, including protecting areas in MPAs. The impacts of MPA networks on stocks and profits have already been demonstrated for several fisheries in southern California (Figure 3; [8]). The same approach can be used to evaluate changes to management regulations (e.g., reduced fishery pressure through a size or season limit or transition to rights-based management) and/or the reduction of uncertainty regarding the status of the stock (e.g., collecting more data to conduct a more sophisticated stock assessment). Such applications can generate a range of viable options for achieving sustainability from which the most efficient action or combination of actions can be selected.

The basic approach presented here, which involves evaluating the likely future effects of MPAs on fishery sustainability, could in principle apply to any fishery in the world and could be used to provide sustainability credit. Perhaps the best candidates are unassessed coastal fisheries, many of which occur in the developing world; and these are the very fisheries that are most difficult to certify using conventional stock assessments. These fisheries compose a huge fraction of global fisheries, but a relatively small fraction of global catch. Applying the MPA certification approach to only this class of fisheries could give rise to substantial local benefits in many parts of the world, but would not have dramatic impacts on the global food system. Many high-volume fisheries, composed of migratory species, demersal species, forage fish, and others, can also benefit from MPAs, though larger protected areas would be required.

Of course, not all fisheries supply the developed-world markets in America and Asia that seek sustainability guarantees. For example, many fisheries in the developing world are used for subsistence and local markets, although international fish processing and distribution businesses increasingly target developing-world fisheries and provide the infrastructure for this seafood to enter more lucrative developed-world markets. It should also be noted that even fisheries that are primarily harvested to produce fish meal and fish oils (e.g., anchovy fisheries) are interested in obtaining certification because of consumer demand for farmed fish that are raised on sustainable feed.

## Looking Forward

We have argued that providing credit for MPAs could significantly increase the incentive to implement MPAs. While it is hard to know how many more MPAs would have been implemented had this practice been adopted at the inception of seafood certification, we do note that, until recently, most regions of the world have had negligible area protected from fishing in MPAs. As the conservation and fishery benefits of MPAs become better known, a growing number of MPAs have been established. This trend is expected to continue, given international commitments and NGO initiatives to expand the global network of MPAs, and could accelerate still further if certification programs were to recognize MPAs. Two prominent examples of MPAs that clearly could have received credit in certification schemes are the Great Barrier Reef Marine Park in Australia, which protects about a third of the reef with no-take areas (with an additional third protected from destructive bottom trawling fishing techniques), and the statewide network of MPAs being implemented in California, which protects about 17% of nearshore waters (with about 10% in no-take areas). Both of these examples show a strong investment in conservation. Yet most of the fisheries in these regions get no credit for these management actions under existing certification programs.

A more explicit integration of MPAs into seafood certification is likely to result in significant benefits. This integration will create more widespread incentives for sustainability, particularly in developing-country fisheries that are often data poor but may have adequate local support for MPA networks. Making certification a more readily attainable goal in parts of the world where it is presently infeasible will enhance the adoption of sustainable practices for harvesting seafood and accelerate the spread of sustainability reforms in ocean management. Furthermore, by providing fishermen with the incentive of certification, support for MPA implementation is likely to increase. With broader stakeholder support, protection in MPAs can accelerate to the scale required for more effective global conservation of marine resources.

## Supporting Information

Text S1 **Supplementary methods.**
(PDF)
