# Supplementary material for: Encourage Sustainability by Giving Credit for Marine Protected Areas in Seafood Certification
Source: PLoS Biol. 2013 Dec 10;11(12):e1001730. doi: 10.1371/journal.pbio.1001730 (PMC3858214; doi:10.1371/journal.pbio.1001730)
Supplement: Text S1 — Supplementary methods. (PDF) [file pbio.1001730.s001.pdf]

Title: Encourage sustainability by giving credit for Marine Protected Areas in seafood certification

Authors: Sarah E. Lester[1,2,*], Christopher Costello[2], Andrew Rassweiler[1], Steven D. Gaines[2], Robert Deacon[3]

Affiliations:
[1] Marine Science Institute, University of California, Santa Barbara, CA 93106-6150.

[2] Bren School of Environmental Science and Management, University of California, Santa Barbara, CA 93106.

[3] Department of Economics, University of California, Santa Barbara, CA 93106.

*Correspondence to: lester@msi.ucsb.edu.

Text S1: Supplementary Methods

To illustrate how mathematical models can be used to compare different strategies for moving a fishery towards certification, we consider the California sheephead (*Semicossyphus pulcher)*, a commercially and recreationally targeted species that has been reduced to 20% of its unfished biomass [1]. We use an existing spatially explicit bioeconomic model of a population of this species in the Southern California Bight [2] to examine several strategies for increasing its biomass. In the model, we break the region into 135 patches, with the carrying capacity of sheephead in each patch is determined by the abundance of shallow hard substrate. Within each patch we simulate an age structured subpopulation; subpopulations are linked by dispersal occurring during the larval phase, with the probability of larvae being produced in one patch dispersing to each other patch based on models of regional ocean currents [3,4]. The model also simulates fishing behavior, distributing a given total fishing effort (i.e. number of boat-days of fishing) across the patches based on the assumption that each fisher seeks to maximize their individual catch per unit effort. MPAs can be incorporated within this framework by closing

some patches to fishing. In that case the fleet model allocates fishing among the open patches based on the same assumptions. The simulation continues until both the fish and the distribution of fishermen have reached a stable state (this eventual equilibrium is not sensitive to initial conditions, provided a non-zero initial population). Parameters and a full description of the model are given in Rassweiler et al. [2]. For this example we use a compensation ratio of 4 and a cost parameter of 0. We emphasize that this is a realistic but somewhat simplified model, and so these results should not be taken as a specific recommendation for sheephead management in southern California. However, the model is closely related to ones that have guided other management decisions in Southern California [5] and is sufficient to demonstrate the power of the approach.

We used the model to examine the stock biomass outcome of three management strategies: 1) MPAs only, 2) MPAs and reduced fishing effort, and 3) MPAs and reduced fishing effort and a larger size limit (in all cases examining a range of MPA coverage from 0 to 100%). We assume that certification requires the biomass to be at least 40% of unfished levels. To model the MPA-only scenario, we found the level of fishing effort that would reduce the biomass to 20% of unfished levels, given no MPAs and the current size limit. We then reran the simulation for different MPA configurations using the same fishing effort. In each simulation MPAs were placed in randomly chosen locations; for each level of MPA coverage 1000 different configurations were tried and the average equilibrium biomass across those trials was plotted as the expected effect of that MPA coverage (Fig. 2). We then repeated this procedure, reducing total effort by 20%. This could reflect new limits on the number of boats, the time spent fishing or other non-spatial regulations that reduced total fishing intensity. Note that this action increases biomass even in the absence of MPAs, and that this increase is evident across the whole range of

MPA coverage. Finally, we examined a case where effort is reduced, as described above, and the size limit was increased by 10%. This change in the size limit effectively protects young sheephead from fishing for an additional year, allowing them more time to reproduce before being caught. The effect of these combined regulations is similar to that of a reduction of effort, but results in larger increases in biomass regardless of MPA coverage.

References:

1. Alonzo S, Key M, Ish T, MacCall A (2004) Status of the California sheephead (*Semicossyphus pulcher*) stock. California Department of Fish and Game Report.
2. Rassweiler A, Costello C, Siegel D (2012) Marine protected areas and the value of spatially optimized fishery management. Proc Natl Acad Sci USA 109: 11884-11889.
3. Mitarai S, Siegel D, Watson J, Dong C, McWilliams JC (2009) Quantifying connectivity in the coastal ocean with application to the Southern California Bight. Journal of Geophysical Research-Oceans 114: doi:200910.201029/202008JC005166
4. Watson JR, Mitarai S, Siegel DA, Caselle JE, Dong C, et al. (2010) Realized and potential larval connectivity in the Southern California Bight. Can J Fish Aquat Sci 401: 31-48.
5. White JW, Scholz AJ, Rassweiler A, Steinback C, Botsford LW, et al. (2012) A comparison of approaches used for economic analysis in marine protected area network planning in California. Ocean Coast Manag: doi: 10.1016/j.ocecoaman.2012.1006.1006.
